# Virtual Prehabilitation in Patients With Cancer Undergoing Surgery During the COVID-19 Pandemic: Protocol for a Prospective Feasibility Study

**DOI:** 10.2196/29936

**Published:** 2022-05-06

**Authors:** Genevieve Lambert, Kenneth Drummond, Bhagya Tahasildar, Francesco Carli

**Affiliations:** 1 Department of Experimental Surgery Faculty of Medicine McGill University Montreal, QC Canada; 2 Peri Operative Program Department of Anesthesia McGill University Health Center Montreal, QC Canada

**Keywords:** prehabilitation, telehealth, functional capacity, cancer care, exercise, malnutrition, psychosocial support

## Abstract

**Background:**

Since the beginning of the COVID-19 pandemic, preoperative care, also termed prehabilitation, has become increasingly relevant due to the decreasing functional and psychosocial health of patients with cancer, which is a result of the pandemic restrictions. Concurrently, access to telehealth has improved; telehealth comprises all remote care delivery facilitated by information technologies (ie, virtually).

**Objective:**

The aim of this protocol is to describe the rationale and methodology for a major trial investigating the feasibility and safety of multimodal virtual prehabilitation services (ie, teleprehabilitation).

**Methods:**

This single-arm feasibility trial aims to recruit 100 patients with cancer to receive teleprehabilitation throughout their preoperative period. The inclusion criteria are as follows: (1) 18 years of age or older, (2) scheduled for elective cancer surgery and referred by a surgeon, (3) medically cleared by the referring physician to engage in physical activity, and (4) have a good comprehension of the English or French language. Feasibility will be assessed by documenting recruitment, adherence, and retention rates, in addition to patients’ motives for not participating in the trial, low participation, or discontinuation. The secondary outcome of safety will be assessed by reporting program-related adverse events.

**Results:**

The Montreal General Hospital Foundation funded the project in August 2020. The protocol was then approved by the Research Ethics Board of the McGill University Health Centre in January 2021 (ID No. 2021-6730). The first patient was recruited in March 2021, and recruitment is expected to end in September 2022. As of March 2022, 36 patients have been recruited, including 24 who have completed their participation. No adverse events have been reported. Data collection is expected to conclude in November 2022. Data analysis will be performed, and the results will be published by the beginning of 2023.

**Conclusions:**

This trial will provide guidance on the use of telehealth in the administration of prehabilitation services. The trial will provide a large amount of information that will respond to gaps in the literature, as there are minimal reports on the use of telehealth rehabilitation and prehabilitation services among elderly populations and in acute contexts, such as the preoperative period.

**Trial Registration:**

ClinicalTrials.gov NCT0479956; https://clinicaltrials.gov/ct2/show/NCT04799561

**International Registered Report Identifier (IRRID):**

DERR1-10.2196/29936

## Introduction

The COVID-19 pandemic prompted many health disparities globally. Notably, the World Health Organization has warned of the possible postponement or cancellation of cancer surgery and the risk of death of many patients awaiting surgery [1,2]. The potential impacts of the pandemic on both the physical and mental health of patients were previously discussed in editorials and articles [3-6]. Prehabilitation is a novel field of study, whereby the principles of rehabilitation are applied preoperatively to optimize patient health with the hope of minimizing the stress experienced with patients’ surgery and the recovery that ensues. Prehabilitation is multidisciplinary and addresses modifiable risk factors by providing patients with multimodal health interventions, such as exercise programs, nutritional support, and psychosocial support [7]. The literature on prehabilitation is rapidly evolving and has highlighted prehabilitation as a useful clinical tool that might attenuate the anticipated preoperative health deficits associated with a cancer diagnosis and its treatments [8]. For instance, patients with cancer are more prone to be in a catabolic state due to neoadjuvant therapies, which adds to the symptom-related burden of the disease and alters dietary patterns [9]. Management of such comorbidities by prehabilitation has been demonstrated to have potential for improving patient fitness, patient-reported health outcomes, and perioperative outcomes [10-12]. These interventions may be especially applicable in the context of the pandemic in view of the extended waiting times to access surgery [3,4,13].

Teleprehabilitation was defined in a previously published commentary by our group in 2020 [5] as the use of technologies to deliver health interventions to patients prior to surgery. Briefly, the commentary elaborated on the benefits and opportunities of using various technological tools available [14,15]. The commentary suggested that combined systems of videoconferencing and wearable devices may be optimal in providing personalized feedback [5]. Videoconferencing has been proven to be feasible and beneficial [16] for delivering rehabilitation, nutritional counseling, and psychological counseling [17-20]. Furthermore, wearable devices, such as training watches, can provide quantifiable feedback to users, which raises awareness of their daily habits (eg, sleep [21], physical activity [22], or inactivity [23]) in order to improve their lifestyle; this is especially important for this high-risk population. However, there is little to no literature on the subject for elderly populations in an acute context, such as the preoperative period [24], and for multimodal teleprehabilitation interventions.

This proposal has been set up to investigate the use of technologies, such as videoconferencing and training watches [5], to enable our prehabilitation clinic to continue to support people with cancer in a virtual format and deliver safe remote counseling by specialist health care providers. The goal of this study is to demonstrate the feasibility and safety of multimodal prehabilitation administered via videoconference. These outcomes have been targeted due to a lack of process and system outcomes being reported in the literature, specifically regarding multimodal interventions and combined technology systems, which, to our knowledge, are almost absent from the literature [15,24]. We hypothesized that it will be feasible to recruit patients with cancer requiring surgery and administer a 4-week individualized prehabilitation program that is delivered virtually. Further, we hypothesize that less than 5% of patients will experience severe adverse events related to teleprehabilitation interventions.

## Methods

### Ethics Approval

The trial protocol was approved by the Research Ethics Board (REB) of the McGill University Health Centre (MUHC), Montreal, Quebec, on January 5, 2021 (trial identification No. 2021-6730; protocol amendment No. V-04). The trial was registered prospectively on March 16, 2021, on ClinicalTrials.gov (NCT04799561).

### Study Design

The protocol is for a single-arm feasibility study. It involves patients who will receive distance-delivered support for all components of the home-based prehabilitation program following a baseline health evaluation, in addition to 2 months of follow-up postsurgery. The program includes a personalized exercise program, nutrition support, mental well-being consultations, and, if needed, smoking cessation support. The technologies (ie, a tablet and an activity-monitoring watch) will be used to provide personalized counseling and patient education remotely and to assess program adherence. Informed consent will be obtained from all patients, as per the Health Canada regulations enforced by the REB, and will precede any assessments.

### Inclusion and Exclusion Criteria

Patients will be deemed eligible for the study if they are 18 years of age or older, have been referred for elective surgical management of thoracoabdominal cancer at one of the MUHC sites, and have been medically cleared for exercise by their surgeons. The research staff will not contact patients who are anticipated to receive surgical interventions in less than 4 weeks from the referral date.

Prior to recruitment, the medical research team will screen all patients for health conditions that might preclude their participation in the prehabilitation program by reviewing their medical files and, if necessary, contacting them over the phone.

Exclusion criteria for participation in the study are as follows: comorbid medical, physical, or psychological conditions, whereby exercise and oral nutrition is contraindicated; acute or unstable cardiac conditions; American Society of Anesthesiologists physical status class of IV or V; disabling orthopedic or neuromuscular disease; psychosis; dementia; cardiac failure (New York Heart Association functional class of III or IV); severe chronic obstructive pulmonary disease (forced expiratory volume in first second of expiration <50% predicted); end-stage liver or kidney disease; and severe anemia (symptomatic or hematocrit <30%). Patients with poor comprehension of the English or French language will not be recruited.

### Patient Recruitment

The recruitment process will begin at the MUHC following cancer diagnosis by surgical investigators. Surgeons will refer prospective study candidates to the study coordinator in the prehabilitation unit, who will then screen and contact patients and explain to them the nature of the study. If the patients express interest, they will be scheduled for an initial visit at the prehabilitation unit to review the study, provide consent, and undergo a baseline health evaluation. During the initial visit, the coordinator will review the details of the study with the patient, provide clarifications as needed, and acquire informed consent in accordance with good clinical practice guidelines. It will be reiterated that participation in the trial is voluntary, and involvement will not affect the quality of care that patients receive. Following the acquisition of informed consent, the baseline evaluation will be conducted by a physician, exercise physiologist, and dietician.

### Baseline and Follow-up Assessments

Prior to performing the baseline assessment, basic health information will be obtained, including the following: (1) prior medical history, (2) standardized anthropometric measurements (ie, height, weight, and waist and hip circumferences), (3) a bioelectric impedance analysis, and (4) a basic blood test (ie, hemoglobin, albumin, prealbumin, creatinine, C-reactive protein, and B-natriuretic peptide). The baseline assessment will include functional tests, a nutritional consultation, and self-reported questionnaires.

The clinic will have all patients perform a battery of tests, which will be used to assess functional capacity, facilitate the personalization of their respective exercise prescriptions, and help in evaluating progress throughout the continuum of care. The battery of tests will include the following: (1) the 6-minute walk test performed in accordance with the recommendations of the American Thoracic Society [25], (2) the timed up and go test [26], (3) the timed sit-to-stand test [27], (4) handgrip strength measured using a hand dynamometer [28], and (5) a timed unilateral curl test [29].

All patients will receive a nutritional consultation with a registered dietician to review dietary habits, assess their nutritional status and risk of malnutrition, and determine their optimal nutritional intervention. The dietician will consult several measures, such as anthropometric data, a complete 3-day food log, recent blood tests (ie, albumin, C-reactive protein, and hemoglobin A_1C_), and an MUHC abridged Patient-Generated Subjective Global Assessment (aPG-SGA) [30], in addition to functional measures (ie, handgrip strength).

Patient-reported measures are important and are often overlooked in the clinical setting. Therefore, patients will be asked to complete questionnaires that subjectively assess the following: functional capacity (ie, the Duke Activity Status Index [31]), quality of life (ie, the EQ-5D [32]), mental well-being (ie, the Hospital Anxiety and Depression Scale [33]), psychological distress (ie, distress thermometer), physical disability (ie, the World Health Organization Disability Assessment Schedule, version 2.0), and energy expenditure (ie, the Community Healthy Activities Model Program for Seniors [34,35]).

Following the baseline assessment, patients will be provided with a Samsung tablet and Polar training watch; they will also be given access to a 45-minute technical workshop on the different preset apps, utilities, and educational video content available [36]. Patients will receive the multimodal prehabilitation services until their respective surgeries; the prehabilitation services will include a personalized exercise program, nutritional guidance and supplementation, psychosocial counseling, and medical optimization as deemed necessary. The exercise physiologists will monitor and interact with patients remotely with weekly virtual counseling sessions, and they will liaise with the multidisciplinary team to provide updates on patient progress. Therefore, patients will only have to come to the hospital for essential medical appointments. Patients will have assessments performed at four time points: at baseline, 24 hours prior to surgery, 4 weeks after surgery, and 8 weeks after surgery.

### Interventions

#### Overview

The program to be provided to patients is two-fold: the first part consists of individual counseling sessions with different health care professionals, and the second component is a home-based prehabilitation program, whereby patients will be remotely followed by exercise physiologists.

The multidisciplinary prehabilitation team will be available to further support patients and their respective needs (ie, exercise, nutrition, and psychosocial support). If needed, health care professionals will be able to contact patients via the Zoom videoconferencing platform (Zoom Video Communications, Inc) [37], which will be facilitated using the tablet provided. 

**Figure 1:**
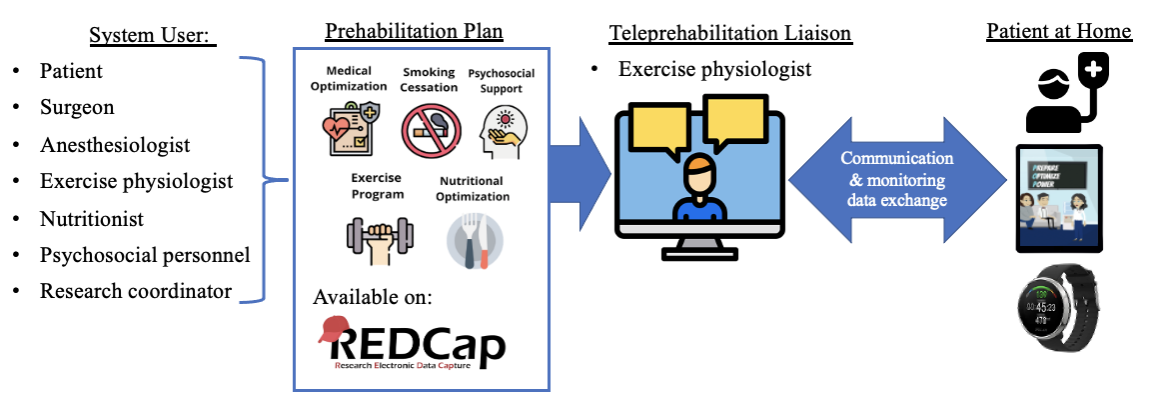
Patient-centered delivery-of-care model.

Exercise physiologists will follow up with patients regularly throughout the continuum of care (ie, before and after their surgery) [38] and refer them to the relevant specialists as needed; see Figure 1 for the patient-centered delivery-of-care plan.

On their tablets, patients will have access to premade educational videos to provide them with further support and guidance on the following important health concepts: physical activity (ie, aerobic, resistance, and flexibility), nutrition optimization (ie, healthy eating, improving protein and energy intake, portion size, and glycemic control), psychological exercises (ie, breathing exercises, relaxation, imaging, and visualization), and smoking cessation.

#### Recommended Exercise Program

As previously discussed, patients’ mobility and capacity to undertake exercise will be evaluated before creating the personalized exercise program. They will be provided with explanations about the program, and their adherence to the program will be monitored and facilitated by the Polar watch provided. Patients will be instructed on how to perform aerobic exercise at home by either walking or cycling (ie, beginning at 50% of their respective heart rate reserve for a minimum target of 30 minutes per day, minimum bouts of 10 minutes, five times per week). The exercise physiologists will adjust the exercise volume or intensity in a stepwise fashion, increasing it by 10% per week, if tolerable. Resistance training will also be encouraged, and patients will be asked to complete a series of eight exercises targeting the major muscle groups three times per week. The exercises will be performed using body weight, elastic bands, or both; two to three sets of 8 to 12 repetitions will be performed.

Physical safety will be addressed before establishing the recommended exercises by evaluating the patient’s functional capacities, prior injuries, and physical limitations. This ensures the safety of every patient in the physical activity program that will be done at home.

#### Nutritional Component: Supplementation and Follow-up

The patient’s nutritional status and dietary intake will be assessed by the nutritionist. The dietician will consult basic anthropometric and laboratory measures, in addition to a nutritional screening tool (ie, the aPG-SGA [30]), in order to help them assess each patient’s dietary needs and extent of nutritional disparity, if present. All patients will receive daily dietary supplements of whey protein. Special precautions will be taken if patients have specific medical conditions (eg, diabetes).

The nutritionist will assess and counsel patients through the videoconferencing app within the first week of enrollment. If needed, additional counseling sessions will be possible through the same platform, depending on patients’ needs. Adherence to the nutritional recommendations will be assessed by having the patient take pictures of their meals one day per week, which will be sent to the dietician.

#### Psychological Component

Patients will be allocated up to 1.5 hours of counseling with a psychology-trained specialist through the teleconferencing app so they can learn mental relaxation and coping mechanisms. Given that patients may experience varying degrees of emotional distress, more sessions can be provided if needed.

#### Lifestyle Modification Component

Patients with smoking habits will meet with a respiratory specialist through videoconferencing. The respiratory specialist will establish recommendations and contact the physician for the recommended smoking cessation protocol. The respiratory specialist will explain the use of an inspiratory muscle training (IMT) device to be used at home. The patient will be instructed to use the IMT device every day for a minimum of two series of 30 breaths each. Weekly improvements will be assessed through home-based videoconferencing.

### Material for Patients

**Figure 2:**
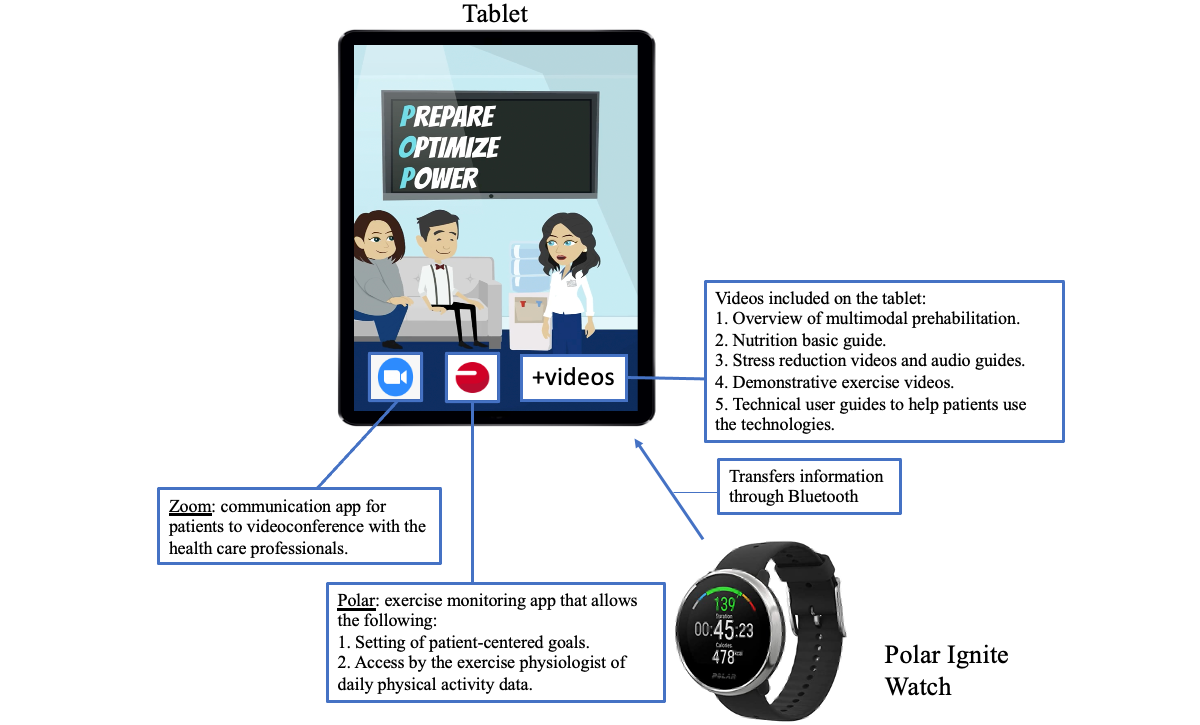
The technological tools of the telehealth system.

In addition to the standard material provided in previous prehabilitation studies (ie, booklet, nutritional supplement, relaxation recording, elastic band, and IMT device), patients in this cohort will have access to two technological tools. These tools will be used to create the telehealth system that allows for interaction between the prehabilitation team and patients during different parts of the program. The two tools, as seen in Figure 2, are (1) a Polar watch and (2) a Samsung tablet, which is loaned to patients for the duration of the study with its respective charger.

The first tool is a Polar Ignite fitness watch. This watch will allow patients to monitor their heart rate and physical activity volume during aerobic and resistance training, in addition to daily activities, directly on their watch or on the app installed on their tablet. For patients to view their physical activity data on their tablet, they must synchronize their watches with the Polar app on their tablet using Bluetooth. It is recommended that they synchronize their watches daily. The information collected from the watch will be uploaded instantaneously onto their Polar personal account when the tablet is connected to the internet. Since patients’ “personal” Polar accounts will be connected to the exercise physiologists’ “coach” accounts (ie, accounts for exercise physiologists to access their patients’ activity logs) prior to provision of the technologies, the daily physical activity information uploaded by the patient will be able to be remotely viewed instantaneously by the prehabilitation team. The information collected (ie, minutes of physical activity, heart rate, and daily step count) will help the prehabilitation team set weekly goals with the patients and monitor their progress.

The second tool is a Samsung tablet. The tablet will create a platform of communication between the patient and the multidisciplinary prehabilitation team. On the tablet, which will be provided with data (ie, internet access), patients will have access to the following: (1) reference videos about the different aspects of their prehabilitation program, (2) a videoconferencing and chat app to communicate with their family and health care professionals (ie, Zoom), (3) a calendar to remind them of counseling appointments, (4) a daily three-question survey concerning adherence, (5) an app to track their physical activity progress, (6) audio recordings for relaxation and coping exercises, and (7) questionnaires and tools for the baseline assessment, preoperative assessment, and postoperative follow-ups. The MUHC information technology (IT) department will format the tablets with their content and apps. This will ensure that all materials will have the same settings with the right security settings applied.

### Outcomes of Interest

The primary outcome is feasibility of teleprehabilitation for high-priority patients with cancer. The criteria to evaluate feasibility are as follows: (1) the recruitment rate, (2) remote access and collection of outcomes measured via technology tools in 70% or more of the patients weekly (eg, step counts and heart rate), (3) an average attendance rate for all components of the multimodal prehabilitation program of 71% or higher (ie, attendance at monitored virtual sessions and independent home-based exercise sessions), and (4) a retention rate of 66% or higher. The rates of recruitment, adherence, and retention will be compared to target thresholds. The recruitment rate is defined as the proportion of eligible patients that consented to participate in the study and is considered to be adequate if greater than 80%. Program adherence is a multifaceted parameter; in this study, adherence will be based on attendance at the synchronous teleprehabilitation sessions and completion of home-based aerobic and resistance exercise sessions that were prescribed. Therefore, adherence to the exercise or teleprehabilitation sessions will be based on the number of sessions completed relative to the number that were prescribed. In a hypothetical example, a patient could be prescribed one teleprehabilitation session, three aerobic sessions, and two resistance sessions per week for a 4-week duration, and they could complete three, 10, and five sessions, respectively. Therefore, their adherence to each component would be 75%, 83%, and 62%, respectively. The total adherence is based on an equally weighted distribution across the above-mentioned components (ie, teleprehabilitation, aerobic, and resistance sessions); therefore, the final attendance score would be 73%. The target threshold for adherence is 71%, which was derived from internal historical data [39]. Retention refers to the number of patients that complete the program with reference to those that consented and is considered to be a success if greater than 66%. Additionally, qualitative measures, such as rationale for refusal to participate, low adherence, and number of dropouts, will be collected to provide more contextual information for interpretation.

The secondary outcome is the safety of delivering telerehabilitation interventions. It will be documented by the occurrence of program-related adverse events, as elaborated by the typology by Ory et al [40] for exercise. The study will be considered safe if the incidence of serious adverse events represents 5% of patients or less. Serious adverse events will be considered as events that would prompt the interruption of the exercise session or the program [41]. The exercise physiologists will question patients on the occurrence of adverse events during weekly synchronous teleprehabilitation sessions. Events will be reported in a timely manner to the physician coinvestigators at the prehabilitation clinic, who will evaluate the severity of the event and the need for temporary or permanent discontinuation of the program, if necessary. Patients will continue to be followed by our research team until the end of the study, even if the exercise program is interrupted (ie, 8 weeks after surgery).

### Statistical Considerations

Descriptive analyses, including means and proportions, will be used to compare demographics and the baseline functional and pathological states of those who remain in the study until completion, as well as those who drop out. This is an essential step to establish that the sample is representative of the population and to mediate potential demographic status biases. Means and SDs will be reported when data are normally distributed, as parametric analyses will be selected. A nonresponse analysis will be conducted using chi-square tests and two-sample *t* tests.

To assess feasibility, the recruitment, completion, and adherence rates will be computed. A one-sample 2-sided *z* test will be performed on the respective rates, with thresholds set at 80% for recruitment and completion and 71% for adherence, based on internal historical data. To assess the adherence rate of individual patients, the completion status of synchronous teleprehabilitation sessions and home-based exercise sessions will be evaluated in a dichotomous manner and will be subject to a proportion of completed sessions that were prescribed. The weekly proportion of completed prescribed sessions will be summed over the whole duration of participation (ie, 4 weeks), and an individual adherence score will be calculated, with equal weights for teleprehabilitation sessions, aerobic sessions, and resistance sessions. Lastly, the individual attendance scores will be pooled with others for the *z* test analysis.

To assess safety, descriptive statistics will be used to report adverse events by type of event (eg, musculoskeletal and cardiac) to establish incidence. The threshold to conclude that teleprehabilitation is safe is a 5% or lower incidence of severe adverse events.

These calculations will be performed using SPSS Statistics for Windows (version 24.0; IBM Corp), with the CI and significance levels preset at 95% and .05, respectively.

### Sample Size and Timeline

The sample size will be based on the evaluation of adherence to the home-based prehabilitation program. In a previous home-based study, we reported an adherence rate to prehabilitation of 78% (n=38) [38] prior to surgery. We assume that the adherence rate in this study will not be much lower because, despite being isolated at home, patients will have access to videos and a watch, which will encourage them to be active and engaged in the program. As per our experience, we expect 20% refusal to participate and 20% loss to follow-up. Approximately 500 patients each year undergo operations at the Montreal General Hospital (MGH) for lung, esophageal, stomach, and colorectal cancer.

### Confidentiality

The technologies in this project (eg, the Zoom videoconferencing platform and the Polar watch app) use confidential information (ie, emails and names). Patients will receive the materials (ie, tablet and watch) with preset accounts from the MUHC IT department, fully coding their identity, where both their names and emails will be coded. Patients will be encouraged to read the privacy notices for both the Polar and Zoom apps. Further, the Polar Ignite watch has a GPS that will record location and distances traveled when activated during cardiopulmonary training sessions. This information will be disclosed to patients, as it may play a role in patients’ willingness to participate.

### Data Collection, Storage, Security, and Handling and Record Keeping

The research coordinator (BT) and the principal investigator (FC) will assign identifiers to each study participant. These ID numbers (ie, PPP1 to PPP100) will not contain any protected health information (eg, social security number and medical record number).

The information collected for the purpose of the research study will be kept strictly confidential and locked in a cupboard within a locked room in the prehabilitation clinic at MGH. All staff, including students, have signed a confidentiality agreement. The password-protected computer where data will be entered is located in a locked room and is not accessible to patients or external members of the hospital.

Since the tablets will be provided with internet data and connection, the cookies will be disabled by the MUHC IT department. Additionally, patients will be asked to close their internet browser after each counseling appointment in order to maximize privacy and data protection.

All information (ie, demographic data, clinical data, patient-reported questionnaires, and adherence data) will be entered and managed in REDCap (Research Electronic Data Capture), a secure clinical trials management system. Data will be stripped of any identifying information (eg, name and address), and each subject will be assigned a study number. All of the information obtained will be treated in a confidential manner.

Study investigators, approved staff, collaborators, and the local REB will have access to review records for research, quality assurance, and data analysis. Data collected before the moment when patients express their wish to stop participating or when patients are lost to follow-up will be able to be analyzed in a confidential manner unless otherwise specified by the patients.

Following the closure of the study, the principal investigator will maintain all study records in a safe and secure location for 7 years, as specified by local regulations. The records will be accessible to allow for appropriate audits and investigations.

## Results

The MGH Foundation funded the project in August 2020. The study recruitment period started on March 12, 2021, with the first patient also recruited in March 2021. As of March 2022, 36 patients have been recruited, including 24 patients who have completed their participation, 6 of whom were unable to complete their 4- and 8-week postoperative evaluations. Currently, there are 12 active participants who are still a part of the teleprehabilitation pathway. None of the participants reported any program-related adverse events. Recruitment is expected to conclude in September 2022, and data collection is expected to conclude in November 2022. Data analysis will be performed, and the results will be published by the beginning of 2023. Following the completion of the study, the results will be communicated in a peer-reviewed journal.

## Discussion

This study is a single-arm clinical trial that aims to investigate the safety and feasibility of delivering teleprehabilitation to patients with cancer who will undergo surgery. There are currently 36 patients recruited, 24 of whom have completed their participation. This is one of the first studies to use technology-assisted interventions in the preoperative setting. Due to the nature of the research, this study may face several challenges and limitations pertaining to (1) the study design, (2) public health measures and disease management, (3) and technologies.

The study design used is inherently subject to various biases due to the lack of a control group or randomization. First, selection bias may be of concern because the study may involve individuals who are more able to participate in the program, especially regarding the exercise component, or who are motivated and, hence, they may be part of a more compliant population. To mitigate the risk of selection bias, we offered participation in the study to all eligible patients who were referred to the clinic, providing technologies for all patients, including those with precarious socioeconomic status. Additionally, to mitigate the risk of misrepresentation of the population, baseline characteristics of patients will be analyzed in order to determine if the observed demographic and exercise capacities are comparable to other trials with surgical oncological candidates. Second, patients involved in the trial may have distinct levels of adherence to the different interventions. To prevent misrepresentation, adherence will be assessed separately for the different components of the program.

The COVID-19 pandemic has impacted the lives of millions of people around the world. COVID-19 pandemic restrictions are still being implemented and will likely remain for the foreseeable future. Upon the new waves due to emerging variants, two potential limits can be foreseen. First, it is possible that patients with cancer may be reluctant to come to the clinic for assessments. To mitigate this challenge, the multidisciplinary prehabilitation team will try to time in-person assessments to coincide with the patients’ other medical appointments. Second, the surgical backlog associated with the first waves of COVID-19 have led to patients missing their window for curative tumor resection of early-grade malignancies [2]. This may present a challenge in prehabilitation, as many patients may experience modifications in their disease management strategies, which can contribute to an elevated dropout rate. To mitigate the misinterpretation of the dropout rate, patients who drop out of the teleprehabilitation program will be asked their motive for doing so.

The technologies in the study constitute another challenge that may limit both data collection and interpretation. There are three technology-related limitations that can be foreseen for data collection. First, patients may not wear the wearable device (ie, sport watch) for the same number of days or hours within a day. Second, the battery in the device could run out. Third, patients could forget to record their training sessions [42]. All of these could impact the validity of the daily measurements and prompt difficulties when pooling the data from different patients together. This challenge of validity can be mediated in two ways. First, the exercise physiologists could be provided with common training on the technologies and the anticipated technical challenges in order to be able to readily provide support to patients. Second, continuous patient feedback could be provided during synchronous teleprehabilitation sessions relating to patients’ real activities if they experienced technological challenges. For example, if a patient has forgotten to stop a training record on their watch, the record may show a 5-hour exercise session, but through the active investigation and open communication between the patient and the exercise physiologist, correction of the erroneous data could be highlighted. Moreover, an additional limitation could be that interpretation of the data can be overwhelming due to the large amount of data collected from each patient daily (ie, multiple time points across the program). This prompts the need for careful statistical considerations when evaluating adherence. To mitigate this challenge, the prehabilitation team will consult a statistician in the conduct of the statistical analyses.

In conclusion, this trial will provide important insights into the use of telehealth in the administration of prehabilitation services. The trial will provide a large amount of information that will respond to gaps in the literature, as there are minimal reports on the use of telehealth rehabilitation and prehabilitation services among elderly populations and in acute contexts, such as the preoperative period [24]. Further, in the context of the evolving pandemic, it can be foreseen that such intervention methodologies will become important tools in providing supportive care to patients with cancer at different institutions in the near and distant future [2].

## Abbreviations

aPG-SGA: abridged Patient-Generated Subjective Global Assessment
IMT: inspiratory muscle training
IT: information technology
MGH: Montreal General Hospital
MUHC: McGill University Health Centre
REB: Research Ethics Board

## Data Availability

The principal investigator (FC) and the research coordinator (BT) will have full access to the final database; data will be available upon reasonable request. The results of the study will be presented in a peer-reviewed journal and international conferences and will be clearly reported on ClinicalTrials.gov.
